# Application of Feedforward Neural Network and SPT Results in the Estimation of Seismic Soil Liquefaction Triggering

**DOI:** 10.1155/2021/1058825

**Published:** 2021-10-18

**Authors:** Tuan Anh Pham

**Affiliations:** University of Transport Technology, Hanoi 100000, Vietnam

## Abstract

Soil liquefaction is a dangerous phenomenon for structures that lose their shear strength and soil resistance, occurring during seismic shocks such as earthquakes or sudden stress conditions. Determining the liquefaction and nonliquefaction capacity of soil is a difficult but necessary job when constructing structures in earthquake zones. Usually, the possibility of soil liquefaction is determined by laboratory tests on soil samples subjected to dynamic loads, and this is time-consuming and costly. Therefore, this study focuses on the development of a machine learning model called a Forward Neural Network (FNN) to estimate the activation of soil liquefaction under seismic condition. The database is collected from the published literature, including 270 liquefaction cases and 216 nonliquefaction case histories under different geological conditions and earthquakes used for construction and confirming the model. The model is built and optimized for hyperparameters based on a technique known as random search (RS). Then, the L2 regularization technique is used to solve the overfitting problem of the model. The analysis results are compared with a series of empirical formulas as well as some popular machine learning (ML) models. The results show that the RS-L2-FNN model successfully predicts soil liquefaction with an accuracy of 90.33% on the entire dataset and an average accuracy of 88.4% after 300 simulations which takes into account the random split of the datasets. Compared with the empirical formulas as well as other machine learning models, the RS-L2-FNN model shows superior performance and solves the overfitting problem of the model. In addition, the global sensitivity analysis technique is used to detect the most important input characteristics affecting the activation prediction of liquefied soils. The results show that the corrected SPT resistance (N_1_)_60_ is the most important input variable, affecting the determination of the liquefaction capacity of the soil. This study provides a powerful tool that allows rapid and accurate prediction of liquefaction based on several basic soil properties.

## 1. Introduction

Liquefaction is the phenomenon in which granular material changes from solid to liquid, with an increase in the water pressure in the pore [1]. Soils that are not drained and subjected to dynamic loads are more likely to liquefy [2]. In geotechnical earthquake engineering, liquefaction and its control factors are important issues [3–6]. In earthquakes, when the pore water pressure reaches the total initial stress level, the increase in the pore water pressure effectively reduces the stress, the soil particles are floating in the water, and then soil liquefaction will occur [7]. Liquefaction is believed to be a major cause of ground failures in earthquakes and a major cause of damage to infrastructure and civil works [8]. Manifestations of liquefaction include reduced soil stress, resulting in loss of bearing capacity [9]. Three types of damage can occur due to soil liquefaction: The first is ground spread and landslide incidents, especially problems with dam embankment [10]. The second is the occurrence of sand blows and lateral spread damage and cracks in the ground [8]. The third is the settlement of the foundation structure of the building, the structure inclination, and the crack of the road surface are serious consequences of soil liquefaction [1]. Therefore, the assessment of the potential for earthquake liquefaction at a site is an important task of earthquake geotechnical engineering.

Scientists have used many different methods to evaluate liquefaction and calculate the factors of safety. Typically, most methods of soil liquefaction assessment are based on the results of the liquefaction and nonliquefaction histories of the soil, combined with in situ or laboratory tests [11–14]. In essence, these methods do not use theoretical calculations but use semiempirical equations. More specifically, the first step is to calculate the cyclic stress ratio (CSR) through the quake's peak ground acceleration (PGA) parameter. Next, the cyclic resistance ratio (CRR) is determined through laboratory cyclic endurance tests, based on undisturbed soil samples taken from the field. Soil is considered liquefied when the CSR value exceeds the CRR value. The disadvantage of this method is that it is difficult to ensure the integrity of the soil samples used. In addition, the high cost of conducting laboratory experiments is an obstacle for this method to be widely applied in practice. A second solution is to use the results of in situ tests such as standard penetration test (SPT) or cone penetration test (CPT) combined with historical observations of liquefaction or nonliquefaction of the soil. From there, curves representing the relationship between CRR and in situ test results are constructed. Since indicators such as SPT and CPT will provide accurate data on soil properties, SPT is considered as a value as a parameter for a more reliable assessment of soil CRR. It has been clearly shown that the method based on the SPT value has a certain range of uses, and beyond this range, the CRR value often proves to be less accurate [15]. In another study, Galavi et al. [16] used the FEM finite element method to describe the behavior of the soil under cyclic load, called the UBC3D-PLM model. The model uses simple plasticity to analyze and predict based on the dynamic set causing liquefaction. The UBC3D-PLM model is a 3D extension of the UBCSAND model which was introduced by Puebla et al. Before that, the first 3D model was proposed by Tsegaye [17] through Plaxis software. However, the limitation of the finite element method is still having to rely on approximate assumptions leading to the existence of errors.

In the last two decades, Artificial Intelligence (AI) is a new method that has been used successfully in several applications in civil engineering [2, 18–20]. In the study of soil liquefaction processes, several AI algorithms have been used and shown to be effective. In their study, Samui and Sitharam [1] used the machine learning technique, namely, Artificial Neural Network (ANN) and Support Vector Machine (SVM), to predict soil liquefaction susceptibility based on SPT. In another study, authors Das and Muduli [21] used Genetic Programming (GP) in an attempt to predict soil liquefaction potential based on CPT data obtained after the Chi-Chi earthquake, Taiwan. In addition, Abbaszadeh Shahri and Maghsoudi Moud [22] developed two Feedforward Neural Network (FNN) models, namely, ICA-MOGFFN and MOGFNN, to determine soil liquefaction potential and showed good accuracy. In general, studies using the machine learning model in general and neural network models, in particular, have achieved certain success in assessing the liquefaction capacity of soils. However, further studies need to be conducted to solve the problems of model performance optimization and model overfitting problem as well as model stability under different sampling methods.

In this study, a machine learning model, namely, Feedforward Neural Network, was developed to predict soil liquefaction potential based on CPT data. A total of 289 datasets from the published literature were used to train and test the model. The model architecture is optimized based on the random search (RS) technique. Then, an ML technique called L2 regularization is used to investigate and solve the model overfitting problem. To evaluate the stability of the model, 300 Monte-Carlo simulations were performed, taking into account the randomness of the data sampling and the initial weights of the model. The paper is structured as follows: the introduction is shown in Section 1.  Section 2 presents the materials needed for the study, including an introduction to the FNN model, L2 regularization technique, RS technique, and performance indicator used in this study.  Section 3 shows the dataset used to develop the FNN models, as well as the statistical information of the input variables.  Section 4 shows the results analyzed by the FNN model and the result of model optimization and compares the final model with some empirical formulas and other ML models to see the outstanding performance of the final FNN models. Finally, Section 5 gives some conclusions and opinions.

## 2. Methods

### 2.1. Feedforward Neural Network (FNN)

FNN is a member of the neural network model family. It can be said that FNN is the first and simplest artificial neural network ever created. In this model, data is transmitted in one direction, from the input layer, through the hidden layer, and to the output layer. In model architecture design, neurons are connected through a value called weight, and each neuron except the input neuron has a fixed bias value. Having to say that, FNN is developed and successful in solving many complex problems of the real world in general and in engineering in particular [23–26]. Moreover, the FNN model can deal with nonlinear relationships between input and output layers through the nonlinear activation functions of hidden neurons. The FNN model needs to be trained before it can be used. The model training aims to find the optimal weights and biases, making it possible for the model to almost accurately predict the actual results. FNN uses many different training techniques, the most common of which is backpropagation.

**Figure 1:**
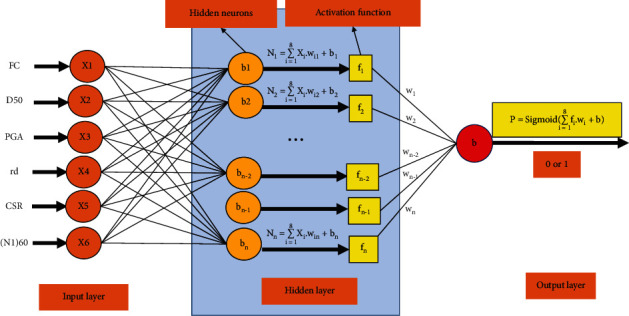
The architecture of FNN used in this study.

The FNN model used in this study can be shown in Figure 1. It can be seen that this model consists of 6 input neurons, some hidden neurons, and a single output neuron. In this research, the binary classification model is used, so the activation function of the output neuron is selected as “Sigmoid”. Since the sigmoid function returns a value between 0 and 1, the model convention is that if the output value ≥0.5, it will be treated as 1 (or True) and otherwise as 0 (or False).

The output value of a neuron in the network can be shown as the following general formula:

(1) $$\begin{array}{c} N_{i}=f\left(\sum_{j=1}^{n}X_{j}w_{ji}+b_{i}\right). \end{array}$$

In which, *f* is the activation function, *X*_*j*_ is the output value of the *j*^th^ node of the previous layer, *w*_*ji*_ is the weight connecting nodes *i*^th^ and *j*^th^, and *b*_*i*_ is the bias of node *i*^th^.

The “Sigmoid” activation function is shown as follows:

(2) $$\begin{array}{c} f\left(x\right)=\frac{1}{1+e^{-x}}. \end{array}$$

### 2.2. L2 Regularization Method

In the field of machine learning, overfitting is the phenomenon where a model is too complex and fits the training dataset and becomes very bad when applied to the test dataset or other new data. It can be said that there are different methods to help the model avoid overfitting. For example, with artificial neural networks, some commonly used methods are regularization [24], early stop, and dropout. Among the above methods, regularization is the solution with high generalization, making the model simpler by decaying the training weights. In this study, the L2 regularization technique is used to minimize overfitting and enhance the predictive performance of the model. In essence, the L2 regularization technique adds a penalty equal to norm 2 of the weights to the model's loss function. The meaning of the L2 regularization technique is to reduce the complexity of the model by prioritizing weights close to zero. In other words, the too large weight will often be eliminated during model training. Therefore, the loss function can be shown as the following formula:

(3) $$\begin{array}{c} f_{c}=J\left(w;X_{\text{train}};y_{\text{train}}\right)+\alpha {\left\|w\right\|}_{2}. \end{array}$$

In which, *J* is the standard loss function of the model; *w* is the weight values; (*X*_train_, *y*_train_) is the training dataset value; ‖*w*‖_2_ is the norm 2 of weight values and *α* is the scale coefficient of the *L*2 regularization technique.

Thus, in the process of model building, determining the appropriate *α* value is very important. Also, *α* = 0 means to remove the *L*2 regularization technique from the training process.

### 2.3. K-Fold CV Technique

**Figure 2:**
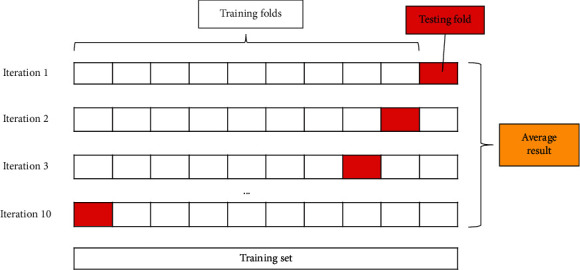
Diagram of 10-Fold CV technique.

To avoid the overfitting problem of the model to the entire dataset, many techniques have been proposed to apply, such as using validation set [18, 19], K-fold CV [26], etc. In particular, the K-fold CV technique is commonly used in machine learning fields when the dataset size is limited. This technique is used to train and modify the model before the model is tested against the final testing set. In this study, the 10-fold CV (Figure 2) technique was applied to confirm the model performance instead of the testing set. In this technique, the training set is randomly divided into 10 different folds, of which 9 folds are used to train the model and the remaining fold is used to validate the model performance. This is done 10 times in order with different validation folds. The final performance of the model is the average of the performance of 10 such runs.

### 2.4. Random Search Method

In a machine learning environment, optimization algorithms are indispensable to enhance performance or find the best model. The family of optimization algorithms can be divided into several categories, such as gradient descent algorithms [27], evolutionary algorithms [18, 28], swarming algorithms [19, 29], and random or grid search algorithms [30]. Among the above optimization techniques, the random search (RS) technique gives simple and good enough efficiency [30, 31].

**Figure 3:**
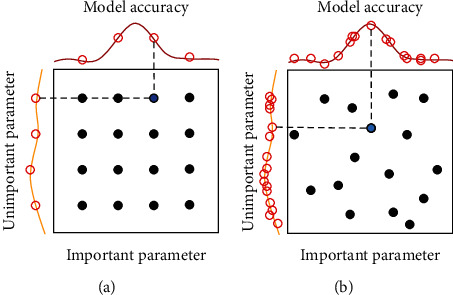
Comparison between (a) grid search and (b) random search for hyperparameters tuning.

The comparison between random and grid search techniques is shown in Figure 3. It can be seen that while grid search combines instances of parameters according to fixed meshes, random search combines those parameters irregularly. Thus, it seems that random search allows the ability to find the optimal combination of parameters better if the number of search combinations is large enough. Many studies have found that random search gives better results than grid search in many specific cases [30, 31]. In this study, the RS method is chosen to select the optimal combination of the most important hyperparameters of the FNN model.

### 2.5. Performance Evaluation

To evaluate the classification performance of the model, various performance indicators are used, including accuracy, precision, and F1. This performance indicator is intended to determine categorical accuracy between forecast and actual results. In general, the higher the accuracy, precision, and F1 values, the more accurate the forecast model. Usually, these values above 0.8 represent a good predictive model, and in the ideal case, these values reach 1 representing the absolute correct prediction model. The formula to calculate these performance indicators is as follows:

(4) $$\begin{array}{c} \text{accuracy}=\frac{\text{TP}+\text{TN}}{\text{TP}+\text{TN}+\text{FP}+\text{FN}}=\frac{\text{number of correct prediction}}{\text{total prediction}},\\ \text{precision}=\frac{\text{TP}}{\text{TP}+\text{FP}},\\ F1=\frac{2\text{TP}}{2\text{TP}+\text{FP}+\text{FN}}, \end{array}$$

where TP = true positive; FP = false positive; TN = true negative; FN = false negative. Meaning of parameter view is in Table 1.

## 3. Data Used

In this study, there is a database of 486 datasets from the published literature, including 288 datasets from the Chi-Chi-Taiwan earthquake [15] and 198 aggregate datasets from other earthquakes [32]. The dataset consisting of a total of 270 liquefied soil samples and 216 nonliquefied soil samples will be used to build and validate machine learning models. All input parameters that may affect the assessment of soil liquefaction are considered [33, 34]. More specifically, the input parameters include peak ground acceleration (PGA), the median diameter of soil particle (D50), shear mass modal participation factor (rd), and the cyclic stress ratio (CSR), and the two input variables related to the cyclic resistance ratio (CRR) are the fines content (FC) and the corrected SPT blow count value (*N*_1_)_60_. The output of the model is whether the soil is liquefied or not (take the values of 1 as liquefied and 0 as nonliquefied).

**Figure 4:**
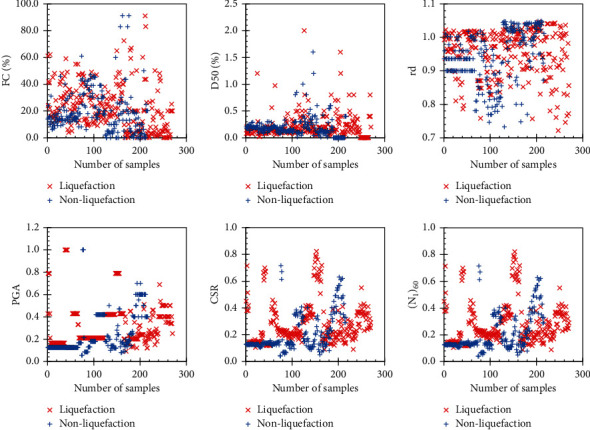
Scatter chart of data used in this study.

**Figure 5:**
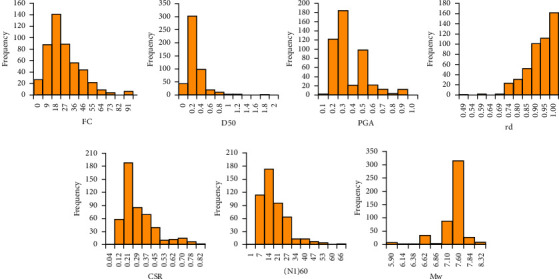
Histogram of data used in this study.

The original dataset was randomly divided into 2 parts: the training part (80% of the dataset) and the test part (20% of the dataset) used to train and validate the performance of the models. The summary of the database statistics is presented in Table 2 which includes the min, mean, max, median, and standard deviation (denoted SD) of all input parameters of the two liquefied and nonliquefied soil history cases used in this study. Besides, the scatter and histogram charts of all the input variables are illustrated in Figures 4 and 5. It can be seen that most of the data are distributed fairly evenly across the range of values. In addition, the data also cover most of the usual values of geological parameters as well as the properties of earthquakes. More specifically, the corrected SPT blow counts (*N*_1_)_60_ range from 0.93 to 65.5. The PGA value of earthquakes is between 0.06 and 1. The fines content of sand is also between 0 and 91 and the shear mass modal participation factor is between 0.49 and 1. The wide distribution of the data used suggests that the model using it is highly generalizable and contributes greatly to the problem of predicting soil liquefaction.

## 4. Result and Discussion

### 4.1. Hyperparameters Tuning

In this section, an FNN model was developed to predict soil liquefaction potential. The FNN model contains many hyperparameters that are important for model training and execution. Therefore, it is necessary to find out the optimal hyperparameters as well as the model architecture. In this study, a set of 5 hyperparameters including the number of hidden neurons, training algorithm, activation function, number of training epochs, and learning rate are considered to be the key hyperparameters of the FNN model. These hyperparameters are searched based on a random search technique of 1000 times. To avoid data leakage, the 10-Fold CV technique was used in this step. At the same time, the testing set was hidden and only used to confirm the performance of the last model.

**Figure 6:**
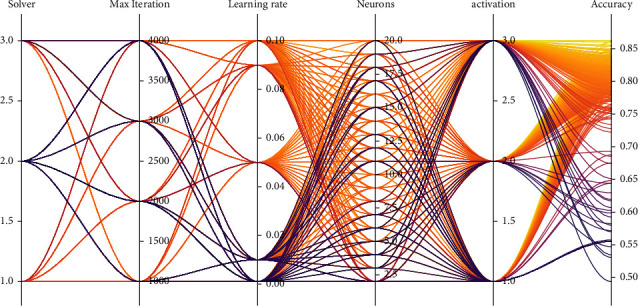
Hyperparameters tuning result.

The permission range of hyperparameters is presented in Table 3. The results of the hyperparameters tuning are shown in Figure 6. Out of 1000 tested models, the model with the best accuracy was selected as the last model. A summary of the best model's hyperparameters is given in Table 4.

It can be seen that the performance of the model shown through the accuracy score changed in a large range, from 0.494 to 0.862. This means that if the wrong set of hyperparameters was chosen incorrectly, the performance of the model can get very poor. Out of all the tested models, the FNN model with the number of neurons equal to 15 and using the “Adam” training algorithm showed outstanding performance on the validation set. In addition, the “ReLU” activation function appears to be consistent with the present data, and with the epoch number equal to 2000, the learning rate should be 0.01 for the model to converge well.

### 4.2. Regularization Model

In the field of machine learning, the selection of good hyperparameters is not sure for the model to achieve high generalization and efficiency in practice. That is because the model is susceptible to overfittings with training data and unable to predict test data or new data well. In this section, the problem of dealing with overfitting using the L2 regularization technique was examined. In it, to find the reasonable penalty value for the fitness value of the FNN model, the parameter *α* takes the values 0, 0.001, 0.01, 0.1, 1, and 10, respectively.

**Figure 7:**
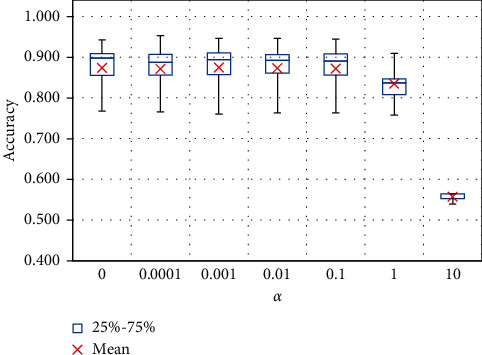
Box plot results using criteria accuracy score.

From the statistical point of view, the comparison of models should consider the randomness of the input factors. Therefore, for each *α* value, the 10-fold CV technique was performed to give the model more generalization to the data. It is important to note that for each alpha value, 300 times random sampling with the 10-Fold CV technique is applied so that the results obtained are more general. Besides, the accuracy score criterion was used as the fitness function for this survey. Results of L2 regularization are shown in Figure 7 and summarized in Table 5.

The results show that when the *α* coefficient is 10, corresponding to the high fitness value penalty, the model does not perform well with low accuracy scores of 0.557. In addition, other alpha values give the model a quite good accuracy, with an average accuracy score ranging from 0.834 to 0.875. Of all the *α* values, the 0.001 value of alpha seems to give the best predictive performance. More specifically, the average accuracy score reaches 0.875 with *α* of 0.001. To improve efficiency and prevent the model from being overfitting with the training data, the *α* value of 0.001 was chosen as the final coefficient of the *L*2 regularization technique in this study.

### 4.3. Capacity of Models

**Figure 8:**
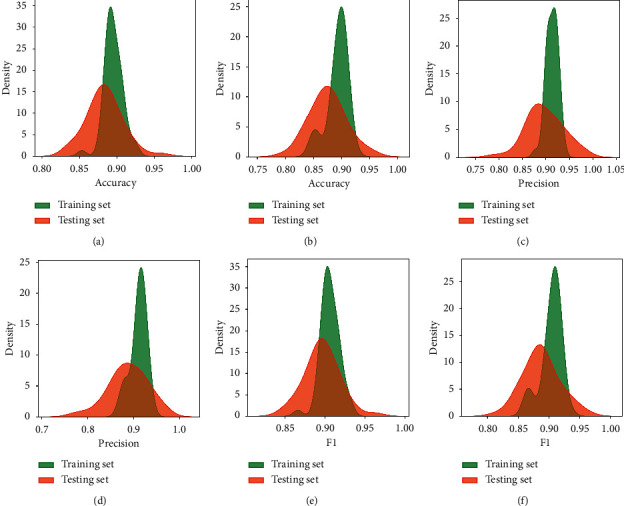
Density diagram of 300 simulations with (a) use regularization (accuracy); (b) no regularization (accuracy); (c) use regularization (precision); (d) no regularization (precision); (e) use regularization (*f*1); (f) no regularization (*f*1).

In this section, the predictability of the final model against new data was confirmed. The two best FNN models, using regularization and not using regularization, were compared. From the statistical point of view, the predictive performance of a model can be greatly influenced by random inputs, such as the random split between training and testing set and random initialization of the weights of the FNN model. Therefore, for a more objective comparison, 300 FNN models with a random sampling between the training and testing set were performed along with random initialization of the model's weights. The results are illustrated in Figure 8 and summarized in Tables 6–8.

The results show that both models provided good performance in estimating the liquefaction capacity of the soil. Out of the two models, the model using regularization gave a better performance on the testing set. To be more specific, the model used regularization achieved average performance criteria, accuracy = 0.884, precision = 0.898, and *f*1 score = 0.897 while the model with no regularization achieved average performance indicators of accuracy = 0.875, precision = 0.888, and *f*1 score = 0.888. Besides, the standard deviation of the regularization model lower than the no regularization model indicated that the first model was more stable. From another point of view, the nonregularization model gave superior results on the training set but does not perform well on the testing set, which proves the model seems to be a bit overfitting and does not achieve high generalization. From the above analysis, model RS-L2-FNN was selected as the final model in this study.

**Figure 9:**
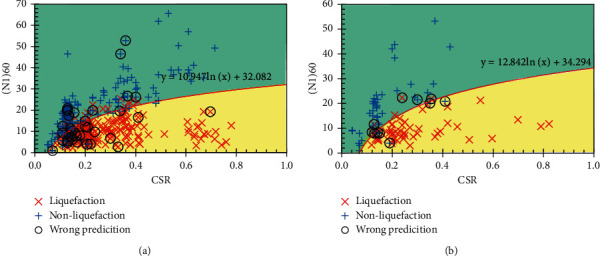
Prediction result of the final FNN model with (a) training set and (b) testing set.

The predictive performance of the final FNN model is shown in Figure 9. It can be seen that the model achieved very impressive performance when it incorrectly predicted 36/388 samples of the training set and 11/98 samples of the testing set.

### 4.4. Comparing with Empirical Formulas and Other Models

In this section, the prediction results of the RS-L2-FNN model are compared with some empirical formulas and ML models. Since soil liquefaction occurs when CSR exceeds CRR, formulas for determining CRR should be considered. More specifically, some experimental formulas for determining CRR are included for comparison as follows:

CRR by Boulanger and Idriss (2016) [34]:

(5) $$\begin{array}{c} {\text{CRR}}_{7.5}=\exp\left[\frac{{\left(N_{1}\right)}_{60cs}}{14.1}+{\left(\frac{{\left(N_{1}\right)}_{60cs}}{126}\right)}^{2}-{\left(\frac{{\left(N_{1}\right)}_{60cs}}{23.6}\right)}^{3}+{\left(\frac{{\left(N_{1}\right)}_{60cs}}{25.4}\right)}^{4}-2.8\right],\\ {\left(N_{1}\right)}_{60cs}={\left(N_{1}\right)}_{60}+\Delta {\left(N_{1}\right)}_{60},\\ \Delta {\left(N_{1}\right)}_{60}=\exp\left[1.63+\frac{9.7}{FC+0.01}-{\left(\frac{15.7}{FC+0.01}\right)}^{2}\right]. \end{array}$$

In which, CRR_7.5_ is the cyclic resistance ratio for an earthquake with a magnitude of 7.5; (*N*_1_)_60*cs*_ is the SPT number of clean sand; Δ(*N*_1_)_60_ is the adjusted SPT increment of clean sand.

CRR by Robertson and Fear (1995) [13]:

(6) $$\begin{array}{c} 100.{\text{CRR}}_{7.5}=\frac{95}{34-{\left(N_{1}\right)}_{60}}+\frac{{\left(N_{1}\right)}_{60}}{1.3}-\frac{1}{2}. \end{array}$$

The adjustment of CRR value for the magnitude of the earthquake is calculated as follows:

(7) $$\begin{array}{c} \text{CRR}={\text{CRR}}_{7.5}.\text{MSF,} \end{array}$$

in which, MSF is the magnitude scale factor; MSF is calculated from Youd et al. (2001) [14] as follows:

(8) $$\begin{array}{c} \text{MSF}=\frac{10^{2.24}}{M_{w}^{2.56}}, \end{array}$$

in which, *M*_*w*_ is the magnitude of the earthquake (*M*_*w*_=7.6 for Chi-Chi earthquake and different values for other earthquakes).

In addition, the multivariate regression (MVR) model is also used to compare with the prediction results of the FNN model. This is one of the most popular models in statistical probability and has proven successful in many different fields [25, 35, 36]. The general formula of MVR has the following form:

(9) $$\begin{array}{c} y_{i}=f\left(x_{1i},x_{2i},\ldots ,x_{6i}\right)=\sum_{j=1}^{6}\beta_{j}.x_{ji}+\beta_{0}, \end{array}$$

where *β*_*j*_ are the coefficients of j^th^ input and *β*_0_ is the intercept.

The least-squares method [37, 38] was used to find the values of the optimal coefficients as follows:

(10) $$\begin{array}{c} S=\sum_{i=1}^{n}{\left(y_{i}-{\bar{y}}_{i}\right)}^{2}⟶\min, \end{array}$$

where *S* is the sum of squared residuals; *n* is the number of training set samples; *y*_*i*_ is the predicted value and ${\bar{y}}_{i}$ is the actual value. It is important to note that ${\bar{y}}_{i}$ only takes 0 and 1 values (corresponds to whether or not the soil is liquefied). Therefore, the value in formula (9) is rounded as follows: if *y*_*i*_ ≥0.5 then *y*_*i*_ = 1; otherwise *y*_*i*_ = 0.

In this study, the optimal coefficients of the MVR model are determined based on the Generalized Reduced Gradient (GRG) optimization algorithm by Lasdon et al. (1978) [39]. The results of the analysis are presented in Table 9.

**Figure 10:**
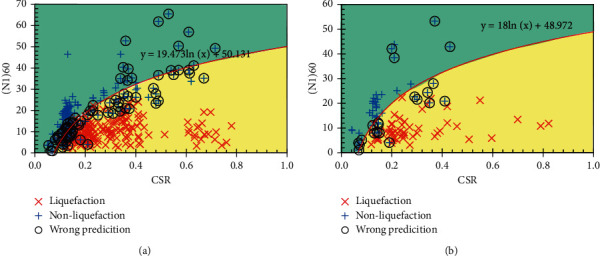
Prediction result by Robertson and Fear: (a) training set; (b) testing set.

**Figure 11:**
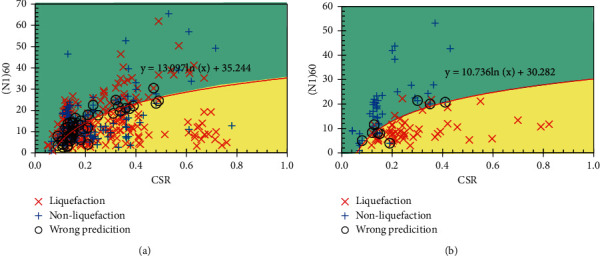
Prediction result by Boulanger and Idriss: (a) training set; (b) testing set.

**Figure 12:**
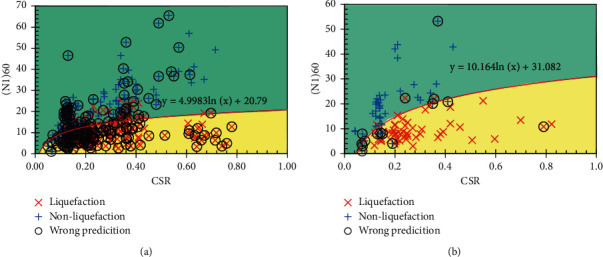
Prediction result by MVR: (a) training set; (b) testing set.

**Figure 13:**
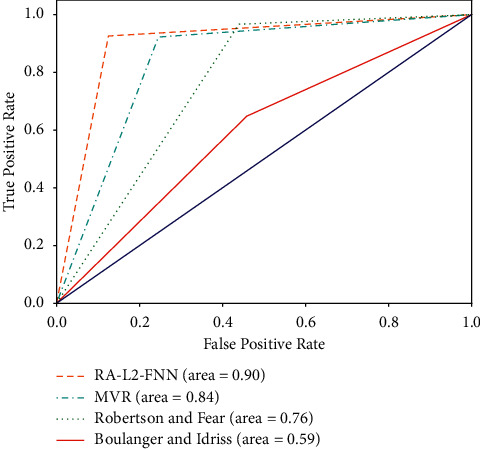
ROC curve of models.

The results of soil liquefaction prediction of experimental formulas and MVR models are shown in Figures 10–12. In addition, the Receiver Operating Characteristic (ROC) curves were built to confirm the classification performance of models as well as the empirical formulas used in this study and are illustrated in Figure 13.

It can be seen that, according to the ROC curves, the RS-L2-FNN model gives the best classification performance with an area value of 0.9, followed by the MVR model with an area value of 0.84. The empirical formula of Robertson and Fear achieves third-order efficiency with an area of 0.76. The experimental formula of Boulanger and Idriss has the lowest efficiency with an area value of 0.59.

Besides, Table 10 presents the comparison of the prediction results of the final FNN model with the empirical formulas, multivariate regression model, and some results of application ML in determining soil liquefaction triggering. It can be seen that most of the empirical formulas give pretty good predictive performance when the accuracy is from 78% to 92%. Some ML models provide a slightly better performance, reaching 93% across the entire dataset. However, it should be emphasized that these datasets are small in size and lack generalizability. The MVR model seems to give a rather low performance as only 54.53% accuracy on the entire dataset. Among the results from those empirical formulas and ML models, the RS-L2-FNN model seems to outperform in predicting only 47/486 data samples, achieving an accuracy of 90.33%. It should be added that the set of 486 data samples was collected from many sources, with different geological conditions and earthquake properties. That shows that the RS-L2-FNN model has the potential to achieve a high level of generalization when it estimates quite accurately the liquefaction potential of the soil.

### 4.5. Sensitivity Analysis

In this study, a global sensitivity analysis technique is performed to evaluate the importance of input parameters on output results using the Monte-Carlo method by Sorbol [41]. The FNN model is built based on the training set and then input data from Saltelli's sampling scheme was used to investigate the correlation between the input and output of the model [42]. Sorbol's global sensitive index is determined by the following formulas:

(11) $$\begin{array}{c} \text{Var}\left(Y\right)=\sum_{a=1}^{6}{\text{Var}}_{a}+\sum_{a<b}^{6}{\text{Var}}_{ab}+\sum_{b<c}^{6}{\text{Var}}_{abc}+\ldots +{\text{Var}}_{abc..6},\\ S_{Ti}=\frac{{\text{Var}}_{a}+\sum_{a<b}^{6}{\text{Var}}_{ab}+\sum_{b<c}^{6}{\text{Var}}_{abc}+\ldots +{\text{Var}}_{abc..6}}{\text{Var}\left(Y\right)}, \end{array}$$

in which Var(Y) is the total variance of the model output; Var_*a*_ is the model output variance in response to variation of the *a*^th^ input variable; Var_*ab*_ is the model output variance in response to the simultaneous variation of the *a*^th^ and the *b*^th^ input; S_Ti_ is the total sensitivity index. *S*_*Ti*_ is in the [0, 1] range, and the larger the value of *S*_*Ti*_, the more important the *i*^th^ input variable.

**Figure 14:**
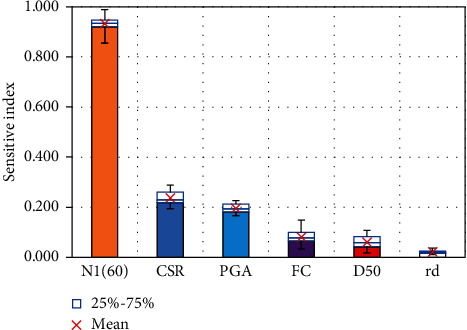
Sensitivity analysis result.

To produce more objective results, 300 simulations were conducted, taking into account the random division between the training and test datasets. The sensitivity analysis result is presented in Figure 14. It can be seen that, among the 6 input variables used to predict the liquefaction triggering of soil, the corrected SPT value (*N*_1_)_60_ was the most important feature when a mean sensitivity index score of 0.933 was obtained. From published studies, it can be seen that the (*N*_1_)_60_ value is decisive to the CRR value, thereby determining the soil liquefaction triggering. The variables PGA, CSR, FC, and D50 were ranked as the second to the fifth important predictors, with an average sensitive index ranging from 0.061 to 0.237. That suggests they influence the results of predicting soil liquefaction, to a certain extent. The remaining input variable rd achieved a low sensitivity value of 0.021, showing that it does not affect the prediction results much.

## 5. Conclusions

In this study, an FNN model was developed to estimate the seismic soil liquefaction triggering. An optimization technique, namely, the RS, was used to choose the optimal architecture for the FNN model and an L2 regularization method was used to solve the overfitting problem of the FNN model.

The results showed that the FNN model with regularization outperforms the nonregularized model. Besides, the L2-FNN models seem to solve well the overfitting problem of base FNN models by demonstrating excellent performance on both training and testing datasets. The results tested through 300 Monte-Carlo simulations showed the superiority and stability of the RS-L2-FNN model compared with the RS-FNN model.

In particular, comparing the estimation results of the RS-L2-FNN model with the results obtained from the empirical formula and some other machine learning models, on the current dataset, shows the outstanding efficiency of the final FNN model. It is necessary to continue synthesizing historical soil liquefaction observations from all over the world, to build an even more generalizable and reliable model.

In addition, the global sensitivity analysis technique was used to detect the most important of the input variables. The results showed that out of 6 input parameters used to estimate the soil liquefaction triggering, the corrected SPT blow counts (N_1_)_60_ were considered the most important feature.

The research results have provided an effective tool in predicting the soil liquefaction triggering and also showed the potential in using optimized machine learning models to replace empirical equations to solve engineering problems.

## Figures and Tables

**Table 1:** Confusion matrix between cluster labels.

Actual	Predicted	
	Positive	Negative
Positive	TP	FN
Negative	FP	TN

**Table 2:** Summary of the statistical information of the database used in the present study.

Unit	FC	D50	PGA	rd	CSR	(*N*_1_)_60_
	—	(%)	—	(mm)	—	—
Count	270	270	270	270	270	270	Liquefied
Min	0	0.00	0.09	0.57	0.09	2.20	
Mean	22.84	0.21	0.32	0.90	0.28	10.04	
Max	91	2.00	1.00	1.00	0.82	25.40	
SD	16.90	0.23	0.19	0.08	0.15	4.79	
Q1	10	0.10	0.20	0.86	0.18	6.60	
Median	20	0.15	0.23	0.92	0.23	8.94	
Q3	31.75	0.24	0.42	0.96	0.35	12.40	

Count	216	216	216	216	216	216	Nonliquefied
Min	0	0.00	0.06	0.49	0.04	0.93	
Mean	19.21	0.20	0.24	0.89	0.21	20.15	
Max	91	1.60	1.00	1.00	0.72	65.50	
SD	15.96	0.19	0.18	0.09	0.14	11.64	
Q1	9	0.10	0.12	0.85	0.12	11.83	
Median	16	0.17	0.16	0.89	0.14	19.21	
Q3	25.44	0.22	0.12	0.90	0.13	20.72	

**Table 3:** Hyperparameters space of machine learning (ML) models.

Hyperparameter	Range
Number of neurons	2 ÷ 100
Solver^(^^*∗*^^)^	1, 2, 3
Activation function^(^^*∗∗*^^)^	1, 2, 3
Max iteration	1000, 2000, 3000, 4000
Learning rate	0.001, 0.01, 0.05, 0.09, 0.1
Cost function	Accuracy
Data used	Training set/10-fold CV

^(^^*∗*^^)^: 1: quasi-Newton method; 2: stochastic gradient descent; 3: Adam. ^(^^*∗∗*^^)^: 1: logistic; 2: tanh; 3: ReLU.

**Table 4:** Optimum hyperparameters of the last FFNN model.

Hyperparameter	Optimum value
Number of neurons	15
Solver	Adam (3)
Activation function	ReLU (3)
Max iteration	2000
Learning rate	0.01
Best accuracy	0.862

**Table 5:** Summary of *L*2 regularization results.

Criteria		*α*						
		0	0.0001	0.001	0.01	0.1	1	10
Accuracy	Min	0.768	0.765	0.760	0.763	0.763	0.758	0.538
	Mean	0.874	0.871	**0.875**	0.873	0.872	0.834	0.557
	Max	0.942	0.953	0.946	0.946	0.945	0.909	0.564
	SD	0.060	0.059	0.061	0.059	0.061	0.047	0.011

**Table 6:** Summary of the 300 simulations using accuracy score.

Model	Dataset	Average	Min	Max	SD
No regularization	Training	0.893	0.845	0.925	0.018
	Testing	0.875	0.796	0.959	0.032

Regularization	Training	0.895	0.853	0.925	0.012
	Testing	0.884	0.837	0.959	0.024

**Table 7:** Summary of the 300 simulations using precision score.

Model	Dataset	Average	Min	Max	SD
No regularization	Training	0.910	0.865	0.941	0.017
	Testing	0.888	0.772	0.964	0.042

Regularization	Training	0.913	0.876	0.940	0.012
	Testing	0.898	0.788	0.982	0.039

**Table 8:** Summary of the 300 simulations using *F*1 score.

Model	Dataset	Average	Min	Max	SD
No regularization	Training	0.904	0.861	0.935	0.016
	Testing	0.888	0.815	0.963	0.030

Regularization	Training	0.906	0.866	0.931	0.011
	Testing	0.897	0.845	0.963	0.022

**Table 9:** The optimal coefficients of the MVR model.

Coefficient	FC	D50	PGA	rd	CSR	(*N*_1_)_60_	Intercept
Value	−0.0045	−0.0089	0.2160	0.4136	1.2103	−0.0364	0.451

**Table 10:** Comparison with other empirical formulas and ML models.

Author	Model	Number of samples	Dataset	Wrong predicted	Accuracy (%)
Seed and Idriss (1971) [12]	Empirical formula	296	Entire dataset	24/296	91.89
Liao and Whitman (1981) [40]	Empirical formula	296	Entire dataset	28/296	90.54
Youd et al. (2003) [14]	Empirical formula	296	Entire dataset	23/296	92.23
Robertson and Fear (1995) [13]	Empirical formula	486	Training set	83/388	78.61
			Testing set	21/98	94.59
			Entire dataset	104/486	78.60
Boulanger and Idriss (2016) [34]	Empirical formula	486	Training set	48/388	87.63
			Testing set	9/98	90.82
			Entire dataset	57/486	88.27
Shahri and Moud (2020) [22]	ICA-MOGFFN	296	Entire dataset	20/296	93.24
	MOGFNN	296	Entire dataset	25/296	91.55
Sarat Kumar Das (2011) [21]	MGGP	227	Entire dataset	30/227	86.78

This study	MVR	486	Training set	206/388	46.91
			Testing set	15/98	84.69
			Entire dataset	221/288	54.53
	RS-L2-FNN		Training set	36/388	90.72
			Testing set	11/98	88.78
			Entire dataset	47/486	90.33

## Data Availability

The processed data are available from the corresponding author upon request.
